# Jejunal Gastrointestinal Stromal Tumor and Gallstones

**DOI:** 10.7759/cureus.53977

**Published:** 2024-02-10

**Authors:** Zaki Busbaih, Insaf Alhazoom, Abdulqader M Albeladi, Alaa Alkhamis, Tayseer Alali, Othman Almohammedsaleh, Ali A Almohammed Saleh, Jawaher I Alraihan

**Affiliations:** 1 General Surgery, Prince Saud Bin Jalawi Hospital, Al-Ahsa, SAU; 2 General and Laparoscopic Surgery, Prince Saud Bin Jalawi Hospital, Al-Ahsa, SAU; 3 Radiology, Prince Saud Bin Jalawi Hospital, Al-Ahsa, SAU; 4 Internal Medicine, Prince Saud Bin Jalawi Hospital, Al-Ahsa, SAU

**Keywords:** cholecystectomy, laparoscopic surgery, gallstones, jejunum, gastrointestinal stromal tumor

## Abstract

Gastrointestinal stromal tumors (GISTs) are considered the most common mesenchymal tumors of the digestive system. However, they make up less than 1% of all GI tumors. GISTs arise from the interstitial cells of Cajal and are commonly found in the stomach and small intestine, and rarely in the colon and esophagus. In this case report, we present a 57-year-old male, a known diabetic, who complained of abdominal pain. He was diagnosed with cholelithiasis and a GIST in the jejunum, which was managed laparoscopically without complications. Most of the time, patients with GIST present with vague symptoms, or sometimes, they are asymptomatic. The most common symptoms are abdominal pain, GI bleeding, and an abdominal mass. These symptoms are usually related to the site of tumor growth, tumor size, and tumor rupture or perforation. Regardless of the tumor location, surgical excision is the gold standard for treating GISTs.

## Introduction

Gastrointestinal stromal tumor (GIST) is considered the most common mesenchymal tumor of the digestive system; however, it makes up less than 1% of all GI tumors. GISTs arise from the interstitial cells of Cajal and are commonly found in the stomach and small intestine, and rarely in the colon and esophagus [1]. The population most affected by GIST is the elderly, with a mean age of 60 years [2]. Based on the tumor location and spread, patients with GIST can either be asymptomatic or present with various clinical symptoms, for example, intestinal obstruction and hemorrhage [3]. Taking into consideration the location, volume, and nature of the tumor, as well as the extent of invasion of surrounding tissues, the preferred and effective treatment for GIST is surgical management [4]. Gallstones in concurrence with GIST are an extremely rare condition. Hereby, we present the case of a 57-year-old male, a known diabetic patient, diagnosed with cholelithiasis and a GIST in the jejunum, which was managed laparoscopically with no complications.

## Case presentation

**Figure 1:**
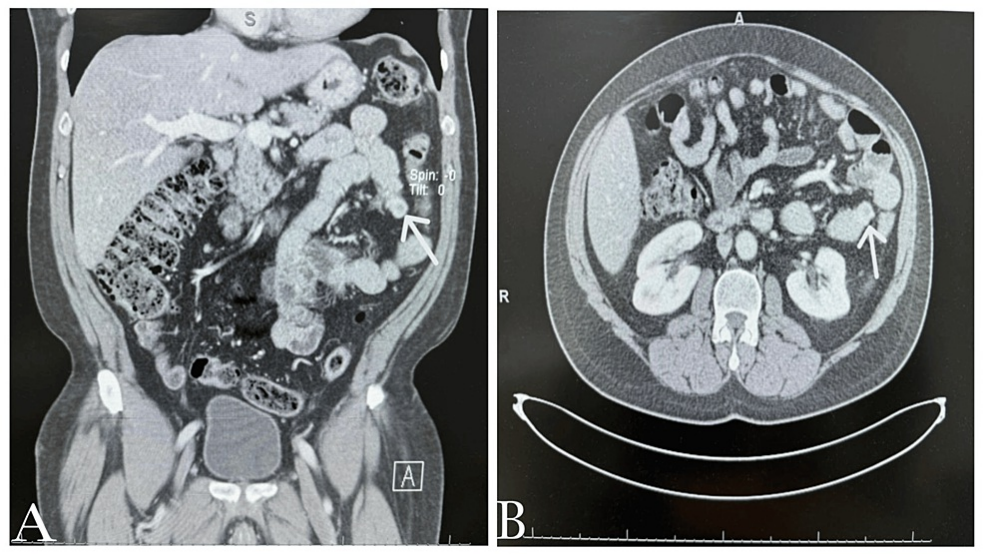
Different views of the tumor in the abdominal CT scan. A) Small tumor (indicated by arrow) in the wall of the proximal jejunum, measuring 2 × 1.5 × 2 cm, in the coronal view of the CT scan. B) Small tumor (indicated by arrow) in the wall of the proximal jejunum, measuring 2 × 1.5 × 2 cm, in the transverse view of the CT scan.

A 57-year-old male, a known diabetic patient with a surgical history of disc surgery eight years ago, presented to the general surgery (GS) clinic, complaining of epigastric pain radiating to the left upper quadrant of the abdomen for one month. The pain increased with eating and was not associated with nausea or vomiting. There was no weight loss or change in bowel habits. On examination, the patient appeared well, conscious, and oriented. Abdominal examination was normal; his abdomen was soft and lax, with no tenderness or rigidity. We ordered some blood tests and imaging for the patient. The lab results, including complete blood count, liver function tests, and renal function tests, were unremarkable, all within the normal range. For imaging, we ordered an abdominal CT scan with IV contrast for the patient. The CT scan revealed an enhancing soft tissue lesion in the wall of the proximal jejunum (2 × 1.5 × 2 cm), which most likely represented a tumor for surgical resection (Figure 1). There was mild diffuse fatty liver infiltration and a tiny gallstone with no cholecystitis. The other abdominal and pelvic organs were within normal limits. There was no free fluid or air and no significantly enlarged lymph node of any size. The major vessels were patent. Skeletal degenerative changes with L4-5 spondylolisthesis and internal fixation hardware were observed. A small fat-containing umbilical hernia was also noted.

**Figure 2:**
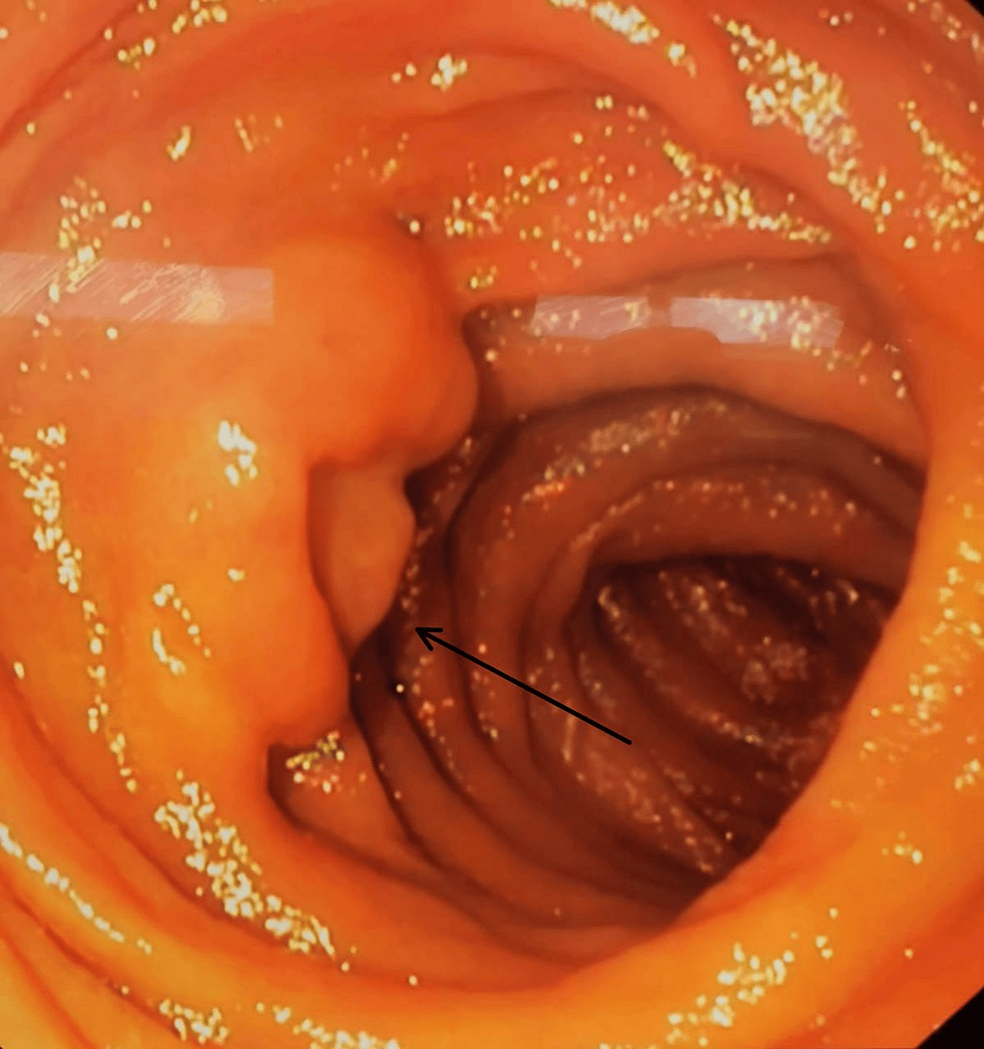
Endoscopic appearance of the tumor in the jejunum.

The management plan for the patient was to perform a GI enteroscopy and then follow up at the GS clinic. Therefore, we arranged with the endoscopy unit, and the patient underwent GI enteroscopy. The enteroscopy results were normal in the esophagus, stomach, and duodenum. In the jejunum, there was a small submucosal lesion (around 1.8 cm) with central depression (Figure 2), but no overlying ulcer was seen at 120 cm from the incisors, and a biopsy was taken.

**Figure 3:**
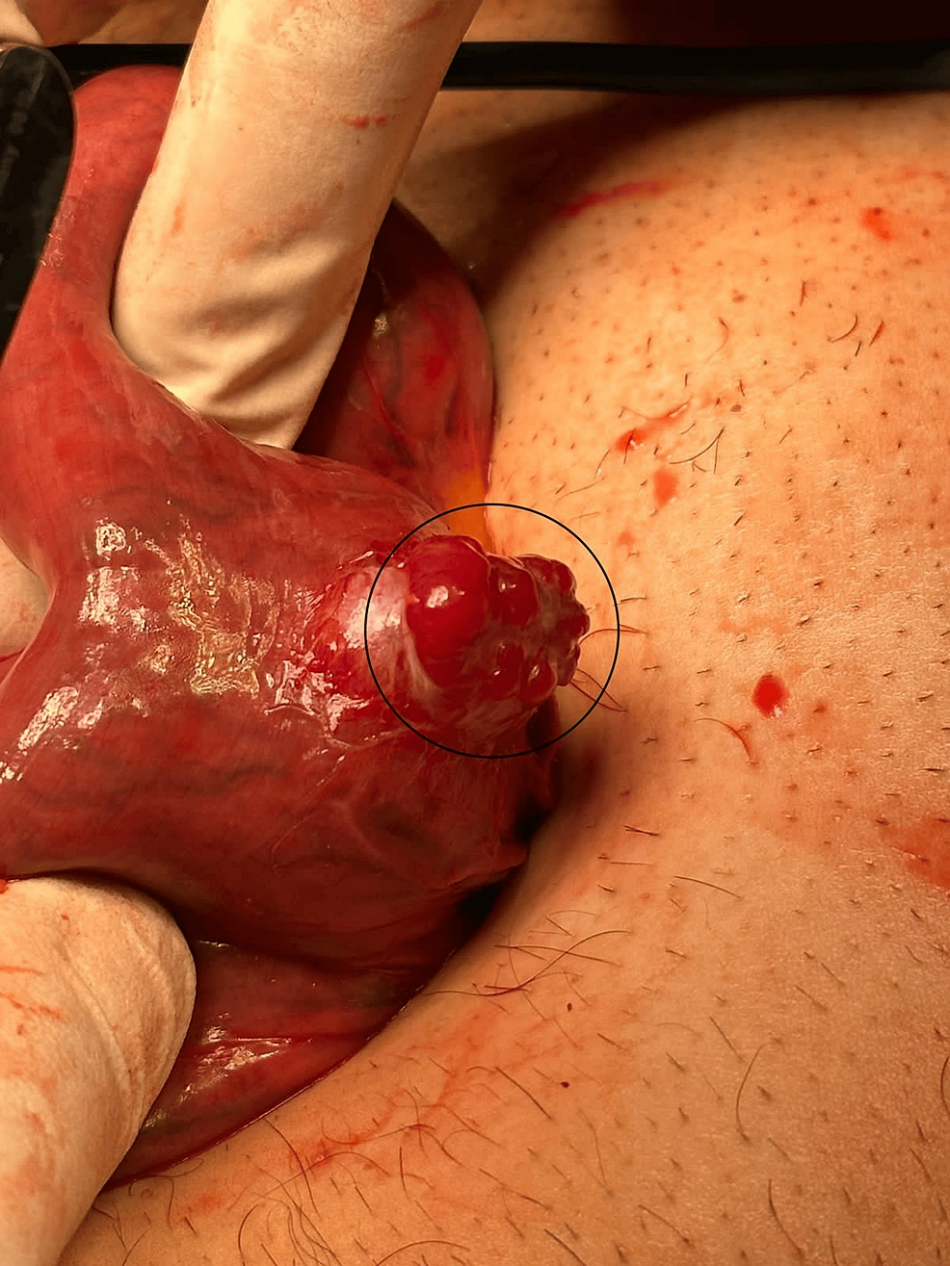
The exophytic mass in the proximal part of the jejunum.

**Figure 4:**
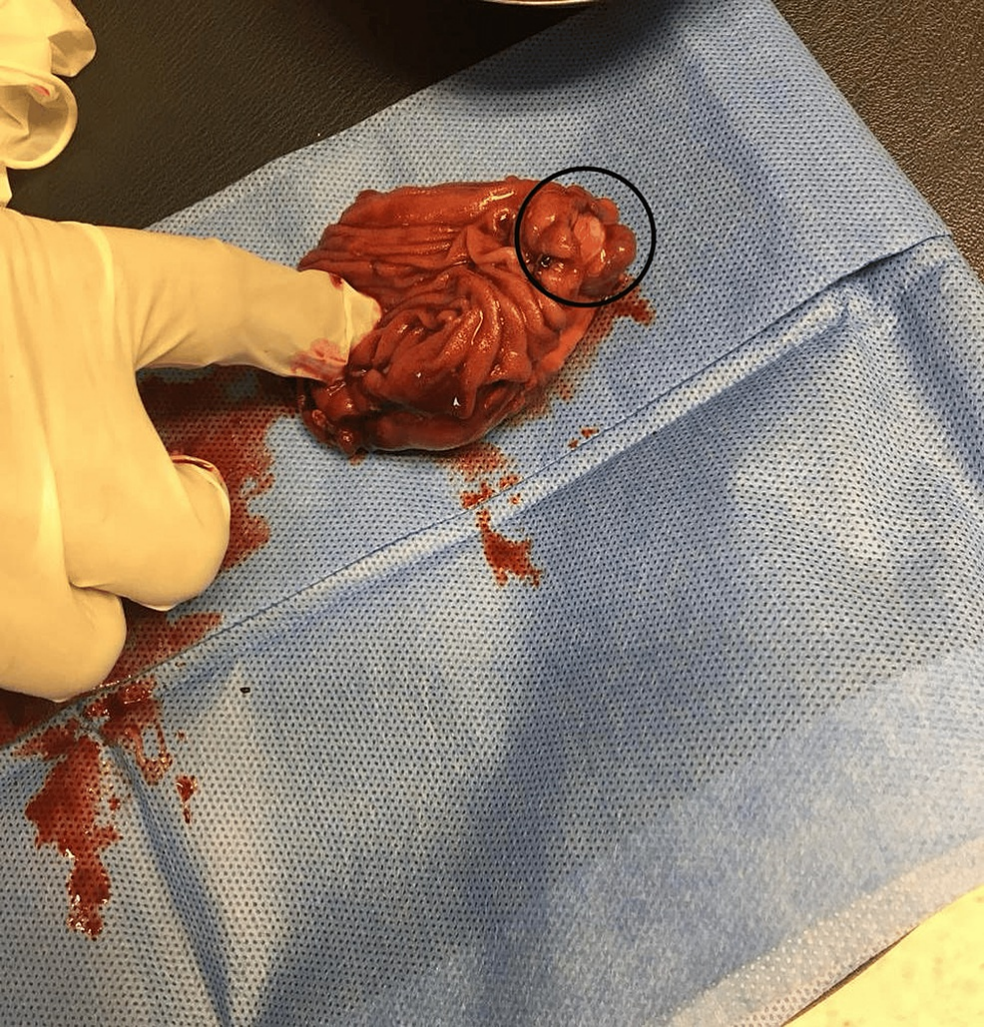
The resected part of the jejunum and the tumor.

After the endoscopy, the patient developed lower GI bleeding and melena, which was managed conservatively, and the bleeding stopped. The patient presented at the GS clinic two weeks after the enteroscopy to follow up on the biopsy result, which showed normal mucosa with attached fibrous tissue. The management plan for his condition was to perform laparoscopic cholecystectomy and jejunal loop excision. The patient was prepared and admitted one day before the surgery. The surgery went well. The patient underwent laparoscopic cholecystectomy and jejunal loop excision during the same procedure. The bowel was run from the duodenojejunal junction to the ileocecal valve laparoscopically. An exophytic mass was found in the proximal part of the jejunum (Figure 3). The umbilical wound was extended by 4 cm, and the segment was delivered through the wound. Resection was done, taking a 10 cm margin proximally and distally (Figure 4), and side-to-side anastomosis was performed using a linear stapler. The segment was returned inside the abdomen, and the procedure was concluded.

After the operation, the patient was doing fine. He was placed on IV fluids, ceftazidime, paracetamol, and tramadol. On day 1 post-operation, we repeated the lab investigations (complete blood count, liver function test, renal function test), and they were normal. The patient stayed in the hospital for three days after the operation. Upon discharge, he was doing well; he was vitally stable, alert, conscious, and oriented. His abdominal examination was soft and lax, with no distension or tenderness. He was discharged on paracetamol and omeprazole tablets. Furthermore, he was given a follow-up appointment (one week) at the GS clinic to assess his condition and review the histopathology results of the gallbladder and tumor.

**Figure 5:**
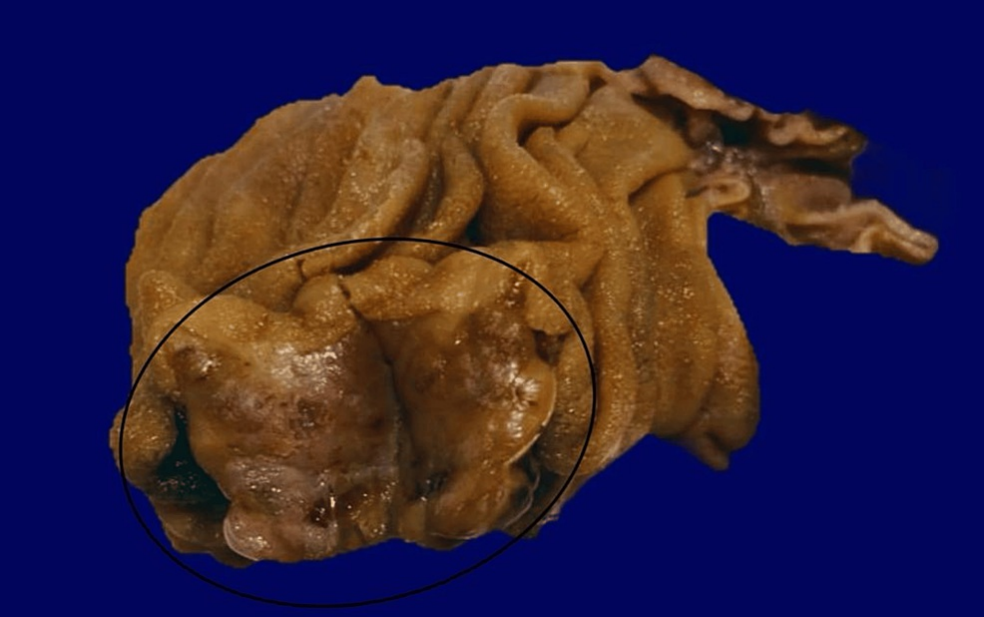
Gross sample of the resected part of the jejunum and tumor, revealing a well-circumscribed, mural, tan-gray mass with overlying intact mucosa.

**Figure 6:**
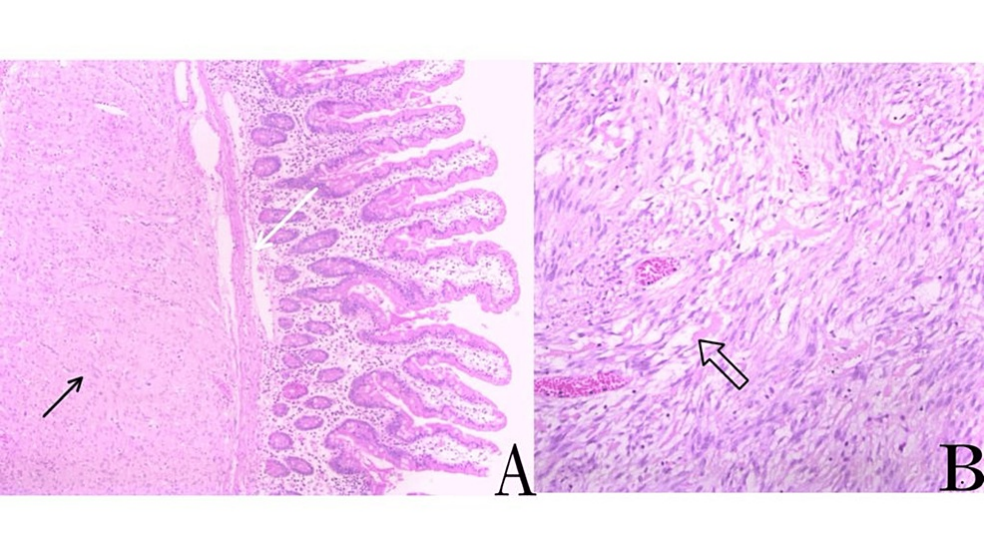
Microscopic examination of the gastrointestinal stromal tumor. A) Intact jejunal mucosa (indicated by a white arrow) with underlying well-circumscribed spindle cell proliferation (indicated by a black arrow). B) Bland, spindled to oval cells with slightly eosinophilic and pale cytoplasm, showing no significant cytologic atypia, mitoses, or necrosis.

**Figure 7:**
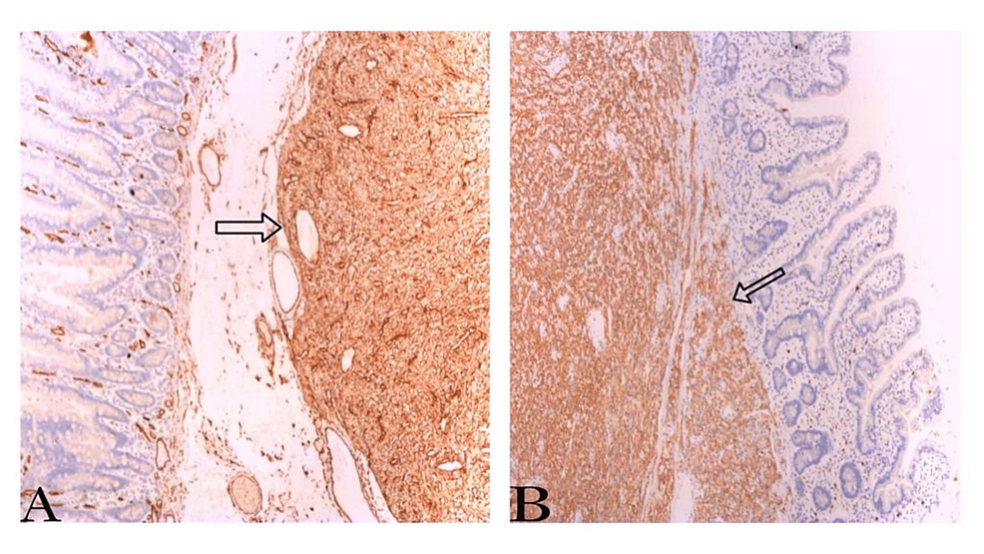
Microscopic examination of the tumor with immunostaining. A) CD-34 immunostaining indicating strong diffuse positivity in neoplastic cells. B) CD-117 immunostaining indicating strong diffuse positivity in neoplastic cells.

The patient presented in the clinic one week after the surgery. He was doing well and had no new complaints. The histopathology result for the gallbladder and the jejunal mass reported that the gallbladder measured 9 x 3 cm. The free external surface was tan, shiny, and smooth, and the wall was 0.3 cm in average thickness. The mucosa was green, velvety, and unremarkable. For the jejunal mass, there was a loop segment of the small bowel (approximately 9 cm in length and 5 cm in diameter). The serosal surface was tan and shiny (Figure 5), and it had a white/gray firm submucosal mass (measuring 2 x 1.8 x 1.5 cm) with a dome-like lobulated projection over the serosal surface. The mass was located 3 cm from the nearest resection margin (distal margin). The remainder of the mucosa was unremarkable, tan, smooth, and shiny. The tumor size was 2 cm, with its histologic type being spindle cell GIST (Figure 6). CD-117, the specific marker for GIST, and CD34, the sensitive marker for GIST, showed strong diffuse positivity in the neoplastic cells (Figure 7). The histologic grade was G1, indicating a low grade. The margin status for all margins was negative for GIST. Thereafter, he was given a follow-up appointment (three months) at the GS clinic for a check-up and to evaluate his condition.

## Discussion

GISTs are the most prevalent mesenchymal neoplasms in the GI tract. However, they represent less than 1% of all GI tumors. Although GISTs can arise anywhere in the GI tract and infrequently affect the extra GI tract, they are typically detected in the stomach or small intestine [5].

Most of the time, patients with GIST present with vague symptoms, but sometimes they are asymptomatic. The most common symptoms they present with are abdominal pain, GI bleeding, and an abdominal mass. These symptoms are usually related to the site of tumor growth, the size of the tumor, and the rupture or perforation of the tumor [6]. As in this case, the patient presented with abdominal pain. When a patient presents with vague symptoms, this makes the diagnosis of GIST rank relatively lower on the differential diagnosis list [7,8].

The diagnosis of GIST depends mainly on radiological imaging, GI endoscopy, or even surgery [9]. In this case, the diagnosis was incidentally made when the patient underwent an abdominal CT which revealed a soft tissue lesion in the wall of the proximal jejunum (2 × 1.5 × 2 cm), indicating a tumor and a tiny gallstone. While the GI endoscopy was mainly normal, there was only a small submucosal lesion (around 1.8 cm) with a central depression, but no overlying ulcer was seen. The findings of the endoscopy are similar to those of most GIST cases. Therefore, the most reliable imaging technique for GIST is contrast-enhanced CT, which can detect the tumor location, size, growth pattern, and blood supply, as well as distant metastases [7,9].

Surgical excision is the gold standard for treating GIST, regardless of the tumor site. The selection of the surgical approach depends on the tumor size and site. For GISTs with a diameter of 5-10 cm, as in this case, laparoscopic surgery is safe and achievable and does not result in an increase in perioperative problems [6]. During laparoscopic surgery, GIST manifests itself grossly as an exophytic growth that resembles a mass, as in this case. Its smooth, gray, and white tone and pseudocapsule further enhance its definition. Necrosis, hemorrhage, and cystic degeneration are infrequently possible [8].

## Conclusions

We presented a patient who complained of abdominal pain, and we discovered that he had a lesion in the wall of the proximal jejunum and a gallstone on imaging. The differential diagnosis list places the diagnosis of GIST comparatively lower when a patient presents with these vague symptoms. The diagnosis of GIST is often made during routine testing, for example, through endoscopy and CT scans. Regardless of the tumor's location, surgical excision is the gold standard for treating GIST.
